# ctDNA Is Useful to Detect Mutations at Codon 641 of Exon 16 of EZH2, a Biomarker for Relapse in Patients with Diffuse Large B-Cell Lymphoma

**DOI:** 10.3390/cancers14194650

**Published:** 2022-09-24

**Authors:** José Díaz-Chávez, Olga Gutiérrez-Hernández, Lucia Taja-Chayeb, Sindy Gutiérrez-Chavarría, Alejandro Avilés-Salas, Myrna Candelaria

**Affiliations:** 1 Research Division, Instituto Nacional de Cancerología (INCan), Mexico City 14080, Mexico; 2 Department of Hematology, Instituto Nacional de Cancerología (INCan), Mexico City 14080, Mexico; 3 Department of Pathology, Instituto Nacional de Cancerología (INCan), Mexico City 14080, Mexico

**Keywords:** EZH2, ctDNA, lymphoma, prognosis, relapse, epigenetics

## Abstract

**Simple Summary:**

It is well known that epigenetic modifications and proteins involved in this process are important in the biogenesis of diffuse large B-cell lymphoma. In this sense, we decided to analyze the EZH2 mutations, which are frequent in this neoplasm, using ctDNA to demonstrate the utility of this tool for searching these mutations. The importance of the study of this gene is due to its role in the biogenesis of lymphomas and also because there are selective inhibitors targeting EZH2. This targeted therapy could be particularly effective in patients with activating mutations in EZH2, remarking the importance of its detection.

**Abstract:**

(1) Background: The epigenetic regulator EZH2 is a subunit of the polycomb repressive complex 2 (PRC2), and methylates H3K27, resulting in transcriptional silencing. It has a critical role in lymphocyte differentiation within the lymph node. Therefore, mutations at this level are implicated in lymphomagenesis. In fact, the mutation at the Y641 amino acid in the EZH2 gene is mutated in up to 40% of B-cell lymphomas. (2) Methods: We compared the presence of exon 16 EZH2 mutations in tumor samples and ctDNA in a prospective trial. These mutations were determined by Sanger sequencing and ddPCR. (3) Results: One hundred and thirty-eight cases were included. Ninety-eight were germinal center, and twenty had EZH2 mutations. Mean follow-up (IQR 25–75) was 23 (7–42) months. The tumor samples were considered the standard of reference. Considering the results of the mutation in ctDNA by Sanger sequencing, the sensibility (Se) and specificity (Sp) were 52% and 99%, respectively. After adding the droplet digital PCR (ddPCR) analysis, the Se and Sp increased to 95% and 100%, respectively. After bivariate analysis, only the presence of double-hit lymphoma (*p* = 0.04) or EZH2 mutations were associated with relapse. The median Progression free survival (PFS) (95% interval confidence) was 27.7 (95% IC: 14–40) vs. 44.1 (95% IC: 40–47.6) months for the mutated vs. wild-type (wt) patients. (4) Conclusions: The ctDNA is useful for analyzing EZH2 mutations, which have an impact on PFS.

## 1. Introduction

Diffuse large B-Cell lymphoma (DLBCL) constitutes the most common of all aggressive types of lymphomas [1]. It is a clinically and molecularly heterogeneous malignant lymphoproliferative disease [2,3]. Traditionally, it has been classified into morphological variants, molecular subtypes, and distinct disease entities. Among no otherwise specified (NOS) cases, an accepted grouping is either the germinal center (GC) subtype or the non-germinal center (non-GC) subtype. The GC subtype has a significantly better prognosis. However, within the GC-subtype, some patients show Myc rearrangement with co-expression of BCL2 or BCL6, defined as double or triple hit lymphomas [2], which have a more aggressive clinical behavior. Recently, the presence of EZH2 mutations has also been implicated in the prognosis of DLBCL [4,5,6].

The epigenetic regulator EZH2 is a subunit of the polycomb repressive complex 2 (PRC2), and methylates H3K27, resulting in transcriptional silencing [7,8]. The overexpression of EZH2 has been identified as a driver in lymphomagenesis [9]. In addition, activating mutations at Y641 amino acid in the EZH2 gene within the EZH2 catalytic SET domain are recurrently and significantly mutated in up to 40% of B-cell lymphomas [10], and particularly in approximately 13–22% of DLBCL [4,11,12]. Unlike wild-type (wt) EZH2, the mutations of Tyr641 (Y641F, Y641N, Y641S, and Y641H) are deficient catalysts of unmodified H3K27 and monomethylation. However, these mutants are superior to the wt enzyme in catalyzing dimethylation, especially trimethylation of histones at H3K27 [13]. Interestingly, Sneeringer et al. demonstrated that EZH2 Y641 mutations, together with wild-type EZH2, lead to higher levels of H3K27 trimethylation, potentiating gene silencing [14]. Given the significance of these alterations in EZH2, several inhibitors, including tazemetostat, have been developed [15].

In this sense, traditionally, mutations are documented in tumor samples as possible targets or prognostic biomarkers. However, with the development of new techniques, the identification of circulating tumor DNA (ctDNA) is available and useful to monitor tumor-specific molecules in the blood, with a sensitivity approaching 1 × 10^6^ cells and also with high tumor specificity [16]. Moreover, access to serial blood samples allows for monitoring these tumor-specific changes and follow-up during treatment [17]. This quantitative approach has been used as a marker for the identification of tumor biology and to predict long-term outcome [18].

Although ctDNA promises as a monitoring tool, the standardization of the collection and processing is necessary to improve DNA preservation and facilitate accurate testing and interpretation of the results. Therefore, in this study, we decided to analyze the activating mutations of EZH2 at Y641 in ctDNA in tumor tissue and peripheral blood, demonstrating the utility of the detection as a prognostic marker, which could also be helpful in identifying patients with DLBCL who can benefit from target therapies against EZH2.

## 2. Materials and Methods

### 2.1. Sample Selection

We conducted a prospective cohort, non-interventional study to analyze the feasibility of detecting the presence of exon 16 EZH2 mutations in ctDNA, and we also evaluated the clinical impact of these mutations in response, relapse, and survival in a cohort of patients with DLBCL. We included consecutive patients diagnosed with DLBCL who were attended at the National Cancer Institute (Mexico City, Mexico) between January 2017 till December 2019. The last follow-up was on 31 July 2021. The inclusion criteria were: age older than 18 years, histopathological diagnosis of DLBCL, without previous treatment, and candidate to be treated with R-CHOP. We excluded patients with hepatitis B or C or HIV, as well as those receiving any other treatment regimen.

Clinical variables analyzed were: age, International Prognostic Index (IPI), presence of B symptoms, bulky disease, clinical stage by Lugano classification [19], serum albumin, lactate dehydrogenase levels (LDH), beta2-microglobulin levels, and performance status determined by the Eastern Cooperative Oncology Group (ECOG) score [20]. Histopathologic variables were GC vs. no-GC by Hans nomogram [21], as well as BCL2, BLC6, and MYC expression, and double-hit lymphoma. Briefly, the GC type was defined by the expression of CD10, or CD10 (−), BCL6 (+), and MUM1 (−). Non-GC type was considered if CD10 (−), BCL6 (−), or CD10 (−), BCL6 (+), MUM1 (+). The cut-off value to consider BCL2 positive was 50%, but 30% for BCL6 and 40% for MYC expression. Lymphomas co-expressing MYC and BCL2 or BLC6 were considered double-expressors. Double-hit lymphomas were defined as those with at least 10% of MYC rearrangements present by fluorescence in situ hybridization (FISH), as previously described [22]. Regarding EZH2 status, they were classified as mutated or wild-type. All samples were collected before beginning the treatment.

All patients were treated with 6 cycles of RCHOP regimen: IV rituximab, 375 mg/m^2^ on day 1; IV cyclophosphamide, 750 mg/m^2^ on day 1; IV doxorubicin, 50 mg/m^2^ on day 1; IV vincristine, 1.4 mg/m^2^, with capping at 2 mg, on day 1; and oral prednisone, 100 mg daily on days 1–5. Then, 18fluoro-deoxyglucose Positron Emission Tomography combined with Computer Tomography (PET-CT) was done at diagnosis and at the end of treatment.

Analyzed outcomes were clinical response after chemotherapy, risk of relapse, progression-free survival (PFS), and overall survival (OS). All patients signed informed consent.

### 2.2. Nucleic Acid Extraction

Tumor samples: 4 sections of the tumor area of 8 to 10 µm from each patient were used. FFPE samples were processed with the AllPrep^®^ DNA/RNA FFPE Kit Cat. No. 80234 (Qiagen, Hilden, Germany) according to the manufacturer’s instructions. DNA was quantified with nanodrop and stored at −20 °C. Peripheral blood: 2 mL of plasma was used, as specified by the manufacturer’s instructions. The ctDNA was extracted using QIAamp Circulating Nucleic Acid Kit (50) Cat. No. 55114 (Qiagen, Germany). The amount of extracted ctDNA was quantified with a Qubit Fluorometer using the Qubit™ dsDNA HS Assay Kit Cat. No. Q32851 (Thermo Fisher Scientific, Waltham, MA, USA) following the manufacturer’s instructions.

### 2.3. PCR Amplification

PCR reactions were performed in a total volume of 25 μL containing 50 ng of DNA, 1 μmol/L of each primer (forward: 5′-ATCTATTGCTGGCACCATCT-3′ and reverse: 5′-CCAATCAAACCCACAGACTTAC-3′), 200 μmol/L dNTPs (Applied Biosystems, Foster City, CA), 2mM MgCl2, 0.25U TaqPol (Applied Biosystems, Foster City, CA, USA) and buffer 1X provided by the manufacturer. PCRs were performed in a 2700 Thermal cycler (Applied Biosystems). The amplifications were done as follows: initial denaturation at 95 °C for 5 min and a final extension at 72 °C for 5 min; denaturation at 95 °C for 30 s, annealing for 30 s at 58 °C, and extension was done at 72 °C for 30 s for 40 cycles. Amplification was verified by gel electrophoresis.

### 2.4. Sanger Sequencing

The PCR products were sequenced in at least two independent amplification reactions to analyze the presence of mutations in exon 16 of EZH2, using the reverse primer: 5´-CCAATCAAACCCACAGACTTAC-3′ (Integrated DNA Technologies; Standard desalted purification synthesis). PCR amplicons were purified using isopropanol precipitation. According to the manufacturer’s instructions, the purified DNA was diluted and cycle-sequenced using the ABI BigDye Terminator kit v3.1 (ABI, Foster City, CA, USA). Sequencing reactions were electrophoresed in an ABI3500 genetic analyzer. Electropherograms were analyzed, and the sequences obtained were compared with the EZH2 reference sequence (GenBank NG_032043.1).

Genomic DNA from the DLBCL cell lines SU-DHL-6, SU-DHL-10, and Pfeiffer were used as controls for the EZH2 Y641N, and Y641F mutations, and wt, respectively.

### 2.5. Droplet Digital PCR (ddPCR)

Those samples of ctDNA that showed a discordant result after Sanger sequencing were analyzed by ddPCR. The tumor DNA was considered the standard of reference.

We amplified 75 bp of EZH2 (exon 16) using primers 5′-TGAATACAGGTTATCAGTGC-3′ and 5′-TCAAAGATCCTGTGCAGA-3′ (Integrated DNA Technologies; Standard desalted purification synthesis) and used Custom PrimeTime^©^ Mini LNA probes (Integrated DNA Technologies) utilized for each of the four most commonly found somatic mutations at this hotspot: Y641H (5′-/56-FAM/AGAA+CA+CT+GTGGAGAGGTA/3IABkFQ/-3′); Y641N (5′-/5HEX/AGAA+AA+CTGT+GGA+GAGGTA/3IABkFQ/-3′); Y641F(5′-/56-FAM/AGAAT+TCT+GTG+GAG+AGGTA/3IABkFQ/-3′; Y641S(5′-/56-FAM/AGAA+T-CCTGT+GGA+GAGGTA/3IABkFQ/-3′) and also one probe targeting the wild-type allele (5′-/56-6-FAM/AGAA+TACTG+TGGA+GAGGTA/3IABkFQ/-3′; + states for Locked Nucleic Acid or LNA bases); these probes were previously designed and reported [23].

The reaction mixture for ddPCR contained 10 μg of ctDNA, 250 nmol/L forward and reverse primers, 250 nmol/L FAM-labeled wt probe, 250 nmol/L HEX-labeled Y641N, 250 nmol/L FAM-labeled Y641S, Y641H, and Y641F probe, and 11 μL of 2 × ddPCR Supermix for Probes (BioRad Laboratories, Pleasanton, CA, USA). Next, distilled water was added to achieve a final volume of 22 μL. The reaction mixture was then partitioned into nanoliter-sized droplets using QX200 Droplet Generator TM (BioRad Laboratories), in which the target DNA was randomly distributed into the droplets. Then, the droplets were transferred to a 96-well plate for PCR reaction in a thermal cycler (Biorad). The PCR program was initiated and held at 95 °C for 10 min, followed by 39 cycles at 94 °C for 30 s, 58 °C for 1 min, and 98 °C for 10 min. The PCR product from each well was then subjected to the QX200 Droplet Reader (BioRad Technologies), which analyzed the fluorescence of each droplet individually using a two-color detection system. Custom software (QuantaSoft; BioRad Technologies) was used to count the number of droplets within each gate.

### 2.6. Statistical Analysis

A descriptive analysis was done for demographic and clinical characteristics. Medians and interquartile ranges (IQR) were used as a measure of dispersion. Clinical and histological variables were compared between wt and mutated cases by the chi-squared test and the Student’s *t*-test, as required. The response was evaluated by Lugano criteria [18]. Progression-free survival (PFS) was defined, from the date of diagnosis, until relapse, progression, or the last visit. Overall survival (OS) was defined from the diagnosis date until death or last visit.

Then, ctDNA concentrations were measured and compared by bivariate analysis with the following clinical prognostic factors: LDH levels, clinical stage, IPI score, response to treatment, and presence of relapse.

Results of EZH2 mutations in the tumor sample and ctDNA were compared, considering the tumor sample as the standard of reference. Sensitivity (Se), specificity (Sp), positive predictive value (PPV), as well as negative predictive value (NPV) were calculated. Se was calculated with true positive/(true positive + false negative). Sp was calculated with: true negative/(true negative + false positive). PPV was: true positive/(true positive+ false positive), and NVP was obtained with: true negative/(false negative + true negative).

The Kaplan–Meier method was used to construct survival curves, and the Log-rank test was used for comparisons. The survival curves compared the mutated and wt cases.

The proportionality assumptions and interaction terms were checked in the final models. The SPSS version 23 software (IBM, Corp. Armonk, NY, USA) was used for computations.

## 3. Results

### 3.1. Patients

A total of 138 cases were included; most of patients were male (n = 74, 53.6%), with at least one site of extranodal involvement (n = 87, 63%), and an advanced disease (n = 96, 69.5%). The median (25–75 IQR) age was 60.1 (50.75–70) years. According to the Hans nomogram [21], most were classified as germinal center (GC) (n = 98, 71%). Clinical and tumor characteristics are detailed in Table 1. From all clinical and histological variables, only the presence of advanced clinical stage was statistically significant different between wt and mutated cases. All mutated patients belonged to the GC cell of origin. All patients were treated with six cycles of R-CHOP, and the response was evaluated by standard criteria [20,21].

### 3.2. Analysis of EZH2 Mutations

**Figure 1:**
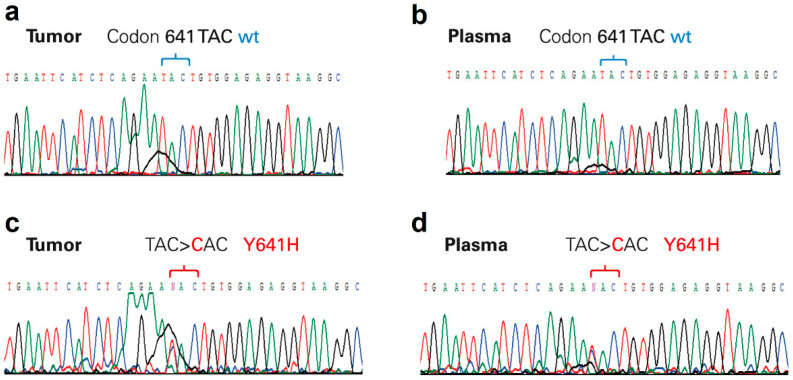
Chromatograms from Sanger sequencing. (**a**) Tumor and (**b**) plasma paired samples with wild-type EZH2-exon 16; (**c**) tumor and (**d**) plasma paired samples with EZH2 mutated at codon 641 (Y641H). The wild-type sequence of codon 641 is TAC.

All cases had tumor and peripheral blood samples and were analyzed for mutations at codon 641 in exon 16 of EZH2. Results revealed that 20 patients of 138 cases with DLBCL (14.5%), corresponding to 20.4% of the 98 GC-DLBCL, had EZH2 mutations, as follows: Y641F (n = 7, 5.1%), Y641N (n = 4, 2.9%), Y641H (n = 3, 2.2%), Y641S (n = 3, 2.2%), I638T (n = 1, 0.7%). Two patients had a double mutation: Y641N + F637L (n = 1, 0.7%), Y641S+Y641F (n = 1, 0.7%). The median concentration (IQR 25–75) of ctDNA was 1555.63 ng/mL (904.0–4410.0). An association was documented between median (IQR 25–75) ctDNA and lactate dehydrogenase (LDH) values: 1085 ng/mL (659–1767) vs. 1920 ng/mL (952–5860) in patients with normal vs. elevated values of LDH, respectively (*p* = 0.01). No association was documented within IPI score, clinical stage disease, response rate or relapse rate, and ctDNA concentration. Regarding ctDNA analysis, after initial Sanger sequencing, only eleven mutations were documented: Y641F (n = 4, 2.9%), Y641N (n = 2, 1.4%), Y641H (n = 2, 1.4%), Y641S (n = 1, 0.7%), Y641C (n = 1, 0.7%), Y641N +F637L (n = 1, 0.7%). Figure 1 presents an example of these chromatograms.

Those patients with a mutation in the tumor, but wt by ctDNA in plasma by Sanger sequencing analysis, were considered false negatives and subsequently were evaluated by droplet digital PCR (ddPCR). After this analysis, nine mutations (previously documented in tumor samples) were confirmed: Y641F (n = 3, 2.1%), Y641N (n = 2, 1.4%), Y641H (n = 1, 0.7%), Y641S (n = 2, 1.4%), Y641S + Y641F (n = 1, 0.7%) (see Table 2). Considering the mutations results in ctDNA by Sanger sequencing, the sensitivity (Se) and specificity (Sp) were 52% and 99%, respectively. After adding the ddPCR analysis, the Se and Sp increased to 95% and 100%, respectively.

### 3.3. Clinical Response

Most of patients achieved complete (70%) or partial (14%) response. Only eleven (16%) progressed during treatment. By bivariate analysis, complete response (CR) was associated with the presence of bulky disease (*p* = 0.033), Lugano clinical stage (*p* = 0.024), and International Prognostic Index (IPI) score (*p* = 0.03). Neither the ctDNA concentration nor the presence of EZH2 mutations was associated with response. After multivariate analysis, only clinical stage remained significant (OR 1.98; 95% IC 1.122–3.378, *p* = 0.02) for response (see Table 3 and Table 4).

### 3.4. Relapse and Survival

**Figure 2:**
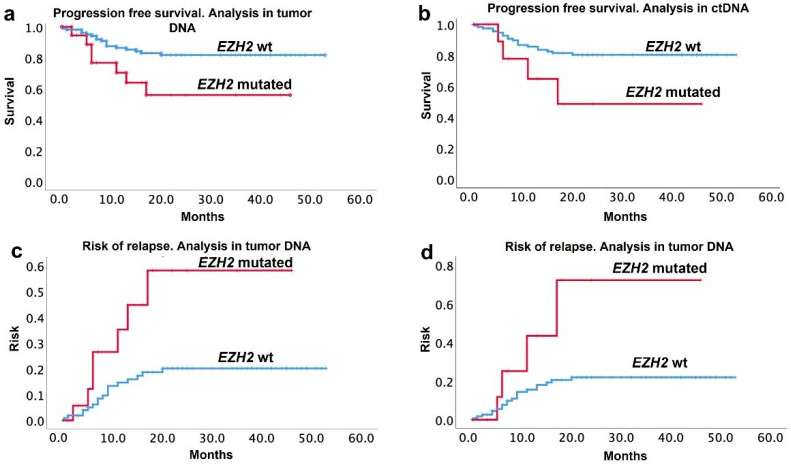
Progression-free survival and risk of relapse curves, according to EZH2 mutation status. Progression-free survival in tumor (**a**) and ctDNA (**b**), and risk of relapse, when analyzing tumor (**c**) and ctDNA by ddPCR (**d**). Blue line = EZH2 wild-type; red line = EZH2 mutated.

The mean follow-up (IQR 25–75) was 23 (7–42) months. During this time, twenty-four patients (17.4%) relapsed. After bivariate analysis, only the presence of double-hit lymphoma (*p* = 0.04) and EZH2 mutations either on tumor analysis (0.047) or ctDNA (0.021) were factors associated with relapse. Median (IQR 25–75) progression-free survival (PFS) was 22.6 (6–40.2) months. The Kaplan–Meier PFS curves of mutated and wt patients in tumor and ctDNA analysis are displayed in Figure 2. The median PFS (95% interval confidence) was 27.7 (95% IC: 14–40) vs. 44.1 (95% IC: 40–47.6) months in the EZH2 mutated vs. wt patients, respectively. The median (95% IC) overall survival (OS) was not different between the mutated or wt EZH2 patients: 40.24 (95% IC: 30.7–49.76) vs. 42.43 (95% IC: 38.67–46.18), respectively.

## 4. Discussion

Epigenetic alterations have been implicated as drivers of lymphomagenesis, particularly EZH2 dysregulation, central to the pathogenesis of DLBCL [9]. Moreover, it has been demonstrated that activating mutations at the Y641 amino acid in the EZH2 gene within the EZH2 catalytic SET domain are frequent in DLBCL and more effective at producing a repressed transcriptional state [11,12]; as a result, there is an increasing interest in developing selective EZH2 inhibitors as a target therapy in lymphomas and other tumors [15]. In this context, the detection of somatic mutations directly from ctDNA is an attractive alternative as a tool to identify patients who can benefit from these therapies, as well as a prognostic marker, because ctDNA is a non-invasive, real-time, tumor-specific biomarker, and therefore an alternative source of tumor DNA for genotyping purposes. In this study, the presence of EZH2 mutations was of particular interest because it was higher in the GC-DLBCL subtype, which may suggest that the search for these mutations could help identify patients with a worse prognosis within the GC subtype.

However, the molecular aberrations within lymphomas are heterogeneous, and different methods are employed for such purposes. In this cohort, the addition of ddPCR analysis to Sanger sequencing increased the sensitivity from 52 to 95%, with a Positive Predictive Value (PPV) of 91% and 100%, respectively.

In healthy subjects, the cell-free DNA (cfDNA) derives from the apoptosis of hematopoietic cells. In lymphoma patients, the total amount of cfDNA has a median concentration of 30 ng/mL of plasma [24,25]. Normal cfDNA needs to be discriminated from ctDNA, and the test used for ctDNA detection and quantification requires the suppression of technical and biological noise in order to achieve the required sensitivity and specificity.

The ctDNA concentrations vary among the lymphoma subtypes, with higher values in diffuse large B-cell lymphoma, Hodgkin lymphoma, and mantle cell lymphoma, and lower levels in low-grade B lymphomas, such as follicular lymphoma [17,26,27,28,29]. In this cohort, the median ctDNA concentrations were within the reported range. Some studies have concluded that higher levels of ctDNA are associated with different prognostic markers, such as advanced clinical stage, poor risk prognostic categories evaluated with IPI score, or survival [30,31,32]. However, in this cohort, we only found an association between ctDNA and elevated levels of LDH; no other prognostic parameter, such as the clinical stage or IPI, correlated with ctDNA concentrations. This difference may be related to the fact that we had very few patients with low clinical stages by low grade, according to IPI score, when compared with other authors. In the same direction, we could not demonstrate a correlation between ctDNA levels and response to treatment or survival since we collected only a sample at diagnosis; however, other authors have indeed demonstrated a correlation between the ctDNA levels and a worse response to treatment, or with survival [31,32,33,34], evaluating the kinetics of ctDNA [31,35,36].

It has been proposed that the liquid biopsy can inform about the whole intratumor heterogeneity. The concordance of results between the analysis in tumor samples, in comparison with ctDNA, may vary with the proportion of a mutation within tumoral tissue and in ctDNA. In this study, the analysis by Sanger sequencing had a very low Se (52%). However, the addition of a more sensible technique, such as ddPCR, increased the detection of EZH2 mutations in all patients analyzed by this technique. Ultra-deep generation sequencing (NGS) methodologies can identify a range of genetic alterations. For example, the cancer personalized profiling by deep sequencing (CAPP-Seq) is considered a disease-specific selector, covering a set of exonic and intronic regions of known recurrent mutations for a specific cancer setting [26,35,37]. Moreover, ddPCR assays are used to detect mutations, but may not be representative of a fraction of ctDNA unless a targeted mutation is known to be trunk in all lymphoma cases. In this sense, genotyping of ctDNA by CAPP-Seq allows the recovery of 100% of the tumor-confirmed actionable mutations of DLBCL, such as EZH2, MYD88, and CD79B [24,33]. However, it is important to mention that one limitation of the application of these technologies to detect mutations in ctDNA in patients with DLBCL is the cost, and that, up to date, there are no kits commercially available with the most frequent mutations.

In addition, ddPCR also measures absolute quantities by counting nucleic acid molecules encapsulated in defined water-in-oil-droplet partitions [23], and the reported sensitivities to detect XPO1, E57K, EZH2 Y641N, MYD88, and L265P mutations range from 80–100%, with 100% specificity [28,38].

Camus et al. documented the usefulness of ddPCR to quantify recurrent and potentially somatic mutations in ctDNA from 88 patients with DLBCL, including EZH2 Y641 mutations. In addition, this author found a 100% concordance for somatic mutation detection between ddPCR and NGS [38]. In our study, the Se of this approach was 95%, with 100% Sp, and, as has been described [38], no false-positive cases have been documented with this method. On the other hand, Dubois et al. [39] initially reported 22%, and thereafter up to 24%, frequency of EZH2 Y641 mutations in GC-DLBCL, which is slightly higher than initially reported by Morin et al. (n = 18/83, 21.7%) [40]. In our study, we found a similar frequency of EZH2 Y641 mutations (n = 20/98, 20.4%) in the same population.

Different authors have evaluated the clinical impact of EZH2 mutations in DLBCL in tumor samples [11,30,41]. However, recently, only Nagy et al. have used liquid biopsy to evaluate the clinical role of EZH2 mutations by ddPCR; however, this study was in patients with follicular lymphoma, and correlated the variant allele frequency with the volume of metabolically active tumor sites observed on 18F-fluorodeoxyglucose positron emission tomography combined with computer tomography (PET-CT) scans [42]. To our knowledge, this is the first study analyzing the EZH2 mutation using ctDNA to evaluate the frequency and the negative impact on PFS in diffuse large B-cell lymphoma. Recently, it has been demonstrated that cell lines with Y641 mutations are more sensitive to selective inhibitors of EZH2; likewise, preclinical data using tazemetostat, an EZH2 inhibitor, in combination with traditional treatment regimens such as CHOP, have demonstrated potent cytotoxicity in EZH2 mutant cell lines [43]. These results have also been confirmed in clinical studies [40,42]. Furthermore, it will be interesting to determine other actionable mutations of DLBCL in ctDNA, such as MYD88 and CD79B, together with EZH2 mutations in ctDNA, and analyze their impact in response to therapy and other clinical variables.

A personalized approach to cancer diagnosis implies integral tumor profiling for each patient, which might be possible by tracking plasma ctDNA tumor-related mutations. The purpose of studying biopsy specimens may be the selection of personalized anticancer therapy relevant to the mutational profile of the specific tumor. However, on the other hand, the application of the plasma ctDNA analysis allows for the monitoring of disease dynamics and the prescribed therapy effectiveness in order to detect any residual tumor after resection, relapse, or even metastasis within a particular patient [44].

## 5. Conclusions

In conclusion, our data support the implementation in the clinic of the analysis of recurrent somatic mutations such as EZH2 in ctDNA to diagnose early detection of molecular relapse, guide salvage therapy based on molecular targets, and identify molecular resistance mechanisms.

## Figures and Tables

**Table 1:** Clinical and histological characteristics at diagnosis.

	n (%) wt	n (%) Mutated	*p*
Total	118 (85.5)	20 (14.5)	-
Gender Female Male	52 (37.7) 66 (47.8)	12 (8.7) 8 (5.8)	0.18
Mean age (25–75 IQR)	60.46 (50–71)	58.55 (52.5–66)	0.68
ECOG * 0–1 >2	86 (62.3) 32 (23.2)	12 (8.7) 8 (5.8)	0.097
Presence of B symptoms Yes No	51 (37) 67 (48.5)	12 (8.7) 8 (5.8)	0.164
Presence of Bulky disease Yes No	43 (31.2) 76 (55.0)	11 (8.0) 8 (5.8)	0.076
Clinical stage I–II III–IV	40 78	2 18	0.01
IPI score+ Low Intermediate-low Intermediate-high High	38 (27.5) 14 (10.1) 17 (12.3) 49 (35.5)	3 (2.2) 2 (1.5) 3 (2.2) 12 (8.7)	0.38
Cell of origin GC ** Non-GC ***	78 (56.5) 40 (29.0)	20 (14.5) 0	0.008
Double hit	12 (8.7)	3 (2.2)	0.52
Extranodal sites 0–1 ≥2	97 21	11 9	0.33
ß_2_ microglobulin Normal Increased	39 (28.3) 79 (57.2)	6 (4.4) 14(10.1)	0.51

* ECOG: Eastern Cooperative Oncology Group, +IPI: International Prognostic Index, ** GC: Germinal-Center, *** Non-GC: Non-Germinal Center.

**Table 2:** Results and comparative analysis of EZH2 mutations in tumor and ctDNA samples.

	Tumor	ctDNA	ctDNA
Mutation	Sanger sequencing	Sanger sequencing	ddPCR *
	N = 20 n (%)	N = 11 n (%)	N = 9 n (%)
Y641F	7 (5.1)	4 (2.9)	3 (2.1)
Y641N	4 (2.9)	2 (1.4)	2 (1.4)
Y641H	3 (2.2)	2 (1.4)	1 (0.7)
Y641S	3 (2.2)	1 (0.7)	2 (1.4)
I638T	1 (0.7)	-----	n.d.
Y641N + F637L	1 (0.7)	1 (0.7)	n.d
Y641S + Y641F	1(0.7)	-----	1 (0.7)
Y641C	-----	1 (0.7)	n.d.

* ddPCR was done only in the false negative cases after Sanger sequencing. n.d.: not determined. -----: not detected.

**Table 3:** Bivariate analysis of factors influencing clinical response.

Factor		% Response	*p* *
Bulky	Yes	48.1	0.03
	No	66.2	
Lugano clinical stage	I–II	71.2	0.024
	III–IV	54.1	
Molecular type	GC **	68.1	0.24
	Non-GC ***	66.0	
IPI score	I–II	75.4	0.03
	III–IV	48	

* *p* = Statistically significant, if <0.05. ** GC: Germinal Center. *** Non-GC: Non-Germinal Center.

**Table 4:** Multivariate analysis of factors influencing clinical response.

Factor	Risk	95 % Confidence Interval	*p* *
ECOG (>2)	1.95	0.572–1.33	0.42
B symptoms	1.26	0.324–1.740	0.44
Bulky disease	1.27	0.526–1.560	0.95
Lugano Clinical stage (III–IV)	1.24	1.21–1.266	0.022
Cell of origin (Non-GC) **	1.38	0.96–1.54	0.58

* *p*: Considered statistically significant, if <0.05. ** Non-GC = Non-Germinal Center.

## Data Availability

All the data presented in this study are available in this article.
